# Trends in deaths and disability-adjusted life-years of stroke attributable to high body-mass index worldwide, 1990–2019

**DOI:** 10.3389/fneur.2023.1211642

**Published:** 2023-06-30

**Authors:** Xiucai Guo, Junxiao Li, Xueyan Yin, Ziping Zhang, Qiongqiong Zhong, Feng Zhu

**Affiliations:** ^1^Pharmaceutical Department and Central Laboratory, Guangzhou Twelfth People’s Hospital, Guangzhou, China; ^2^Department of Public Health and Preventive Medicine, School of Medicine, Jinan University, Guangzhou, China

**Keywords:** stroke, disease burden, obesity, body mass index, disability-adjusted life-years, prevention

## Abstract

**Background:**

High body mass index (HBMI) is an independent risk factor for stroke. Previous studies on the incremental burden of the rapid growth of stroke attributable to HBMI are incomplete and lag behind. We aim to assess the global burden of stroke attributable to HBMI based on a public database online.

**Materials and methods:**

Study data were taken from the Global Burden of Disease, Injuries, and Risk Factors Study; deaths, the Disability-Adjusted Life-Years (DALYs), and their age-standardized rates were screened. The join point regression was used, wherein age-standardized rates were referred to as temporal trends in disease burden.

**Results:**

Deaths from stroke attributable to HBMI worldwide were on the rise during 1990–2019, with an increase of 88.75%. Age-standardized DALYs were on the rise during 1990–2003 but declined during 2003–2013, with a turning point in 2013 and an increasing trend since then [*the Annual Percentage Change* (*APC*) = 0.30%, *p* < 0.05]. China, India, Indonesia, the Russian Federation, and the United States of America shared in sequence the rate of leading deaths and DALYs in 2019. The Socio-Demographic Index (SDI) was associated with an increasing trend in age-standardized deaths (*R* = −0.24, *p* < 0.001) and age-standardized DALYs (*R* = −0.22, *p* = 0.0018).

**Conclusion:**

A range of indicators for the global burden of stroke attributable to HBMI have been on the rise for *the* past three decades. Tremendous efforts worldwide should be in place to control and treat stroke attributable to HBMI, especially in regions with high-middle and middle SDIs and among middle-aged and aged populations.

## Introduction

Stroke is one of the leading causes of mortality and morbidity worldwide, with a series of terrible data on patients, deaths, and age-standardized rates of the Disability-Adjusted Life-Years (DALYs); it is also the third-leading cause of death and disability combined in populations of all ages and the second-leading cause of the DALYs in populations aged ≥50 (1, 2). *The estimated total global direct and indirect costs of stroke in 2017 were approximately 1.12% of the global gross domestic product (GDP)* (3). High Body-Mass Index (HBMI), which is a controllable factor for overweight and obesity, is known to indirectly increase the risk of stroke by inducing hyperlipidemia, hypertension, diabetes, and other diseases (4). *Typically used to define overweight and obesity in epidemiological studies*, *HBMI has been labeled a pandemic due to its continued growth worldwide for over 2 decades*. Obesity is a major risk factor for Non-Communicable Diseases (NCDs), including cardiovascular disease, cancer, chronic respiratory disease, and diabetes (5). *Populations of overweight or obesity in the world have doubled since 1980 to the extent that nearly a third of the world’s population is now classified as overweight or obese* (6), *and the number* reached 3 billion in 2019, wherein more than 5 million deaths were attributable to various causes of obesity, and among *those* were adults aged ≥20 (Organization). Previous studies showed that HBMI was associated with a variety of cardiovascular outcomes, *but the effects of HBMI on the risk of stroke remain varied* (7) *although the risk of stroke was positively correlated with HBMI and the association was stronger in men and ischemic stroke* (8). *Current estimates of the global burden of stroke attributable to HBMI and its temporal trends are sparse*. *Accurate and up-to-date estimates of this burden are important for planning research and the resulting evidence-based strategies for stroke prevention and management*.

*The Global Burden of Diseases*, *Injuries*, *and Risk Factors Study* (GBD) provides an opportunity to incorporate newly available datasets, enhance method performance and standardization, and respond to outbreaks of scientific knowledge. Due to various premature deaths and disabilities by human diseases, a series of scientific assessments for the burden of disease progress and adverse patterns need to be conducted. *We based this study on the newest data from the GBD 2019 to assess the global burden of stroke attributable to HBMI*, *including deaths*, *DALYs*, *and their age-standardized rates*.

## Materials and methods

### Data source and study sample

Study data from 1990 to 2019 were taken from the GBD 2019, a publicly available resource online “http://ghdx.healthdata.org/gbd-results-tool” by the Institute for Health Metrics and Evaluation (IHME) *at the University of Washington*, *the World Health Organization* (WHO (9);), *and members of the Global GBD Collaborative Group*, wherein 369 diseases and injuries, 21 regions, and 204 countries and territories were *recorded*, with a series of newcomers including the Cook Islands, Monaco, San Marino, Nauru, Niue, Palau, Saint Kitts and Nevis, Tokelau, and Tuvalu. *The GBD 2019 is an ongoing effort*, *which is updated annually and allows for consistent comparison over time from 1990 to 2019 by age and sex*, *as well as across locations*. *It also produces standard epidemiological and summary measures and can be estimated from life tables*, *estimates of prevalence*, *and disability weights*. *The GBD study is performed in compliance with the Guidelines for Accurate and Transparent Health Estimates Reporting* (*GATHER*) *guidelines for reporting health estimates*.

### Stroke identification

*Each cause and related states cover the years 1990 to 2019 and were identified with standard case definitions*. *Stroke was defined by the WHO criteria and was estimated based* on the International Statistical Classification of Diseases and Related Health Problems (ICD), the GBD 2019, *and the Cause List Mapped to the ICD codes of the I64 in the 10th ICD* (10) *as follows:* rapidly developing clinical signs of focal (at times global) disturbance of cerebral function lasting more than 24 h or leading to death and of presumed vascular origin, wherein ischemic attack and subarachnoid hemorrhage were included.

### High body-mass index

*We clarified the standard BMI in continents for the World Obesity Atlas 2023 taking the BMI ≥ 25 kg/m^2^ as HBMI*, *which is the acknowledged estimate for global levels of overweight and obesity*. HBMI is defined as overweight and obese with a BMI > 25.00 kg/m^2^. Overweight (or pre-obesity) and obesity are observed in adults with a BMI between 25.00 and 29.99 kg/m^2^ and ≥ 30.00 kg/m^2^, respectively (11). Obesity is a chronic complex disease due to excessive adiposity with the multifactorial risks of obesogenic environments, psychosocial factors, and genetic variants (12).

### Disease burden measurement

*We collected raw data and used analytic tools on the GBD website*. Disease burden was assessed with a range of indicators including deaths, DALYs, *and the age-standardized rates of DALYs and deaths*. Age-standardized rates were calculated with a no-weighted mean of the GBD year’s age-specific proportional distributions for national locations with populations greater than 5 million in the GBD year to update the world population age standard (1). In brief, age-standardized rates were generated from several parameters, including summing up the products of age-standardized rates (*a_i_*, wherein *i* is the *i*th age class), a number (or the weight) of persons (w_i_) in the same age subgroup *i* (a reference of the standard population), and a dividend of summing up standard population weight (13): $ASR=\frac{\sum_{i=1}^{A}a_{i}w_{i}}{\sum_{i=1}^{A}a_{i}}\times 100,000$ . *According to this formula*, *we presented age-standardized deaths per 100*,*000 persons per year and the DALY estimates per 100*,*000 people*, *with the direct method of standardization and WHO’s standard population as a reference* (14). *Additionally*, *204 countries and territories were divided into 21 regions and five regional SDI* (*Socio-Demographic Index*) *groups: high*, *high-middle*, *middle*, *low-middle*, *and low SDIs* (Table 1). *More details on the SDI calculation were addressed in previous studies* (1, 15), *wherein age was divided into three groups: 15–49*, *50–69*, *and ≥ 70*. We present 95% Uncertainty Intervals (UIs) for every metric based on the 25th and 975th ordered values of 1000 draws of the posterior distribution. *Temporal trends are represented by age-standardized DALYs and age-standardized deaths; their increase* (*or decrease*) *and stability are significant* (*p < 0*.*05*) *and insignificant* (*p ≥ 0*.*05*) *for the slope* (*Annual Percentage Change*, *APC*), *respectively; temporal trends here were performed in models of the join point regression* (*the version of 4*.*9*.*1*.*0*), *wherein the Z Test was used to assess a hypothesis on segmentation points and the p values were two-sided*, *with a significance level of 0*.*05*. In brief, when H0 (a segmentation point at 0) was first assumed, a traditional linear regression model could be used; H1 represented a segmentation with at least one point. Once H0 was rejected, the significance between 1 segment point and *n* segment points was assessed. *According to a reference for the APC* (16), time series were built and taken as independent variables, while age-standardized DALYs and deaths were taken as dependent variables. The R software (R core team, version of 4.2.1, Vienna, Austria) was used in the visualization for indicators and used for linear correlations and coefficients of the SDI to age-standardized DALYs and deaths. Statistical significance was defined as *p* < 0.05. A visualization tool (GBD Compare|IHME *Viz* Hub^1^) was used to draw maps online.

**Table 1:** Deaths, DALYs, and their corresponding age-standardized rates in the years 1990 and 2019.

		Deaths				The DALYs			
		No. ×10^5^ (95% UI) in 1990	Age-standardized no. ×10^−5^ (95% UI) in 1990	No. ×10^5^ (95% UI) in 2019	Age-standardized no. ×10^−5^ (95% UI) in 2019	No. ×10^5^ (95% UI) in 1990	Age-standardized no. ×10^−5^ (95% UI) in 1990	No. ×10^5^ (95% UI)in 2019	Age-standardized no. ×10^−5^ (95% UI) in 2019
									
Global		5.78 (2.97–9.18)	14.49 (7.32–23.31)	10.91 (6.55–15.86)	13.2 (7.93–19.32)	179.44 (96.37–280.67)	421.09 (223.52–660.85)	348.71 (222.71–486.37)	416.62 (265.74–581.11)
Sex									
	Female	3.20 (1.74–4.93)	15.02 (8.10–23.41)	5.26 (3.25–7.58)	12.09 (7.49–17.39)	96.26 (55.28–146.02)	436.96 (249.40–662.82)	165.27 (109.67–227.87)	384.02 (254.9–528.94)
	Male	2.58 (1.21–4.34)	13.62 (6.25–23.06)	5.65 (3.28–8.39)	14.27 (8.20–21.52)	83.18 (40.44–138.61)	400.06 (192.95–665.05)	183.45 (112.30–261.01)	448.87 (273.35–642.67)
SDI									
	High SDI	1.05 (0.59–1.6)	10.21 (5.73–15.45)	1.05 (0.64–1.55)	5.91 (3.74–8.33)	30.63 (18.22–44.13)	308.41 (184.54–440.11)	32.92 (22.47–44.29)	214.62 (150.79–283.06)
	High-middle SDI	2.44 (1.39–3.63)	22.97 (12.84–34.85)	3.13 (1.92–4.51)	15.54 (9.51–22.43)	70.78 (41.78–102.41)	635.49 (373.92–926.59)	91.46 (59.92–126.44)	459.46 (300.41–632.98)
	Middle SDI	1.42 (0.62–2.55)	13.07 (5.46–23.55)	3.91 (2.29–5.76)	15.25 (8.76–22.97)	48.6 (22.42–84.17)	399.93 (181.92–699.32)	128.57 (79.81–182.39)	479.25 (295.7–684.01)
	Low-middle SDI	0.59 (0.23–1.11)	9.14 (3.49–17.5)	2.04 (1.19–3.01)	14.21 (8.09–21.44)	19.95 (8–37.21)	278.33 (110.82–519.78)	68.35 (41.22–98.04)	446.4 (265.68–645.95)
	Low SDI	0.27 (0.11–0.49)	10.24 (3.94–19.01)	0.77 (0.43–1.15)	13.38 (7.34–20.65)	9.37 (3.87–16.97)	321.41 (130.19–585.22)	27.18 (15.61–39.88)	422.32 (240.73–625.66)
Region									
	Andean Latin America	0.03 (0.02–0.05)	14.79 (8.82–21.31)	0.06 (0.04–0.08)	9.69 (6.17–13.75)	1.23 (0.77–1.71)	492.45 (309.19–686.58)	1.96 (1.34–2.67)	326.09 (220.71–446.17)
	Australasia	0.02 (0.01–0.03)	9.4 (5.42–13.88)	0.02 (0.01–0.03)	4.5 (2.81–6.54)	0.61 (0.39–0.85)	264.49 (168.44–363.32)	0.61 (0.42–0.81)	144.11 (102.56–185.95)
	Caribbean	0.04 (0.03–0.06)	16.12 (9.49–23.61)	0.08 (0.05–0.12)	15.93 (9.8–23.04)	1.41 (0.87–2)	507.05 (311.74–717.64)	2.59 (1.68–3.61)	502.52 (325.2–701.4)
	Central Asia	0.16 (0.1–0.22)	33.34 (20.98–46.53)	0.28 (0.19–0.37)	37.61 (24.48–51.76)	4.91 (3.22–6.61)	982.54 (641.88–1327.29)	8.87 (6.23–11.52)	1060.7 (735.1–1399.77)
	Central Europe	0.51 (0.33–0.7)	34.79 (22.4–47.91)	0.43 (0.28–0.62)	20.46 (13.26–28.8)	14.4 (9.71–19.05)	972.85 (655.16–1285.06)	11.13 (7.61–14.86)	571.43 (400.58–755.39)
	Central Latin America	0.11 (0.06–0.15)	11.91 (6.95–17.35)	0.22 (0.14–0.3)	8.96 (5.59–12.66)	3.79 (2.4–5.23)	374.65 (233.39–523.38)	7.1 (4.75–9.62)	285.57 (189.22–388.11)
	Central Sub-Saharan Africa	0.04 (0.02–0.07)	16.73 (7.55–28.67)	0.09 (0.05–0.15)	15.55 (8.02–25.85)	1.43 (0.68–2.41)	508.55 (242.51–860.06)	3.2 (1.68–5.04)	472.55 (248.52–757.81)
	East Asia	1.11 (0.28–2.42)	12.21 (2.99–26.62)	2.73 (1.22–4.65)	12.98 (5.76–22.47)	35.21 (9.1–75.03)	353.57 (90.11–758.61)	83.87 (39.57–138.28)	393.61 (185.9–649.64)
	Eastern Europe	1.01 (0.65–1.4)	36.34 (22.95–50.71)	1.04 (0.67–1.45)	31.08 (20.17–42.85)	27.6 (18.59–36.78)	978.67 (657.04–1307.66)	28.46 (19.64–37.65)	886.8 (618.11–1162.26)
	Eastern Sub-Saharan Africa	0.1 (0.04–0.18)	11.16 (4–21.69)	0.29 (0.16–0.44)	16.03 (8.8–25.01)	3.42 (1.34–6.37)	358.39 (136.72–668.9)	10.35 (6.03–15.2)	493.81 (281.57–737.98)
	High-income Asia Pacific	0.15 (0.05–0.27)	7.57 (2.65–13.94)	0.12 (0.04–0.21)	2.89 (1.23–4.97)	4.89 (1.82–8.59)	237.1 (87.56–419.46)	3.78 (1.63–6.35)	117.18 (52.09–192.15)
	High-income North America	0.3 (0.18–0.42)	8.62 (5.36–12.03)	0.43 (0.28–0.6)	7.15 (4.81–9.59)	9.73 (6.45–13.02)	297.48 (199.49–394.41)	14.27 (10.1–18.22)	265.67 (193.38–332.71)
	North Africa and Middle East	0.38 (0.24–0.54)	21.03 (12.8–30.06)	0.89 (0.61–1.21)	19.96 (13.26–28.16)	13.67 (9.08–18.65)	658.76 (430.15–907.03)	31.34 (22.55–41.17)	618 (440.07–822.65)
	Oceania	0.01 (0–0.01)	24.92 (12.35–40.97)	0.02 (0.01–0.03)	25.5 (13.55–41.75)	0.35 (0.19–0.55)	870.57 (463.3–1377.05)	0.88 (0.5–1.37)	906.82 (507.7–1411.69)
	South Asia	0.38 (0.14–0.75)	6.26 (2.16–12.47)	1.59 (0.9–2.34)	10.63 (5.92–15.9)	13.03 (4.73–24.97)	187.85 (68.07–364)	53.42 (31.22–77.57)	336.8 (196.44–490)
	Southeast Asia	0.28 (0.1–0.54)	9.62 (3.28–19.05)	1.26 (0.74–1.83)	19.15 (11.03–28.06)	10.51 (3.91–19.65)	330.05 (119.25–627.87)	45.54 (28.23–64.32)	648.74 (395.7–921.88)
	Southern Latin America	0.08 (0.04–0.12)	17.66 (9.58–26.53)	0.09 (0.05–0.12)	10.46 (6.42–14.68)	2.56 (1.41–3.75)	542.57 (299.02–792.49)	2.57 (1.68–3.47)	326.02 (215.54–437.26)
	Southern Sub-Saharan Africa	0.06 (0.04–0.08)	19.67 (13.22–27.1)	0.12 (0.08–0.15)	20.8 (14.57–27.66)	2.11 (1.49–2.76)	640.46 (450.5–844.37)	3.75 (2.82–4.73)	598.8 (443.15–764.31)
	Tropical Latin America	0.25 (0.14–0.36)	24.91 (14.17–36.87)	0.36 (0.25–0.48)	14.6 (9.91–19.58)	8.6 (5.18–12.25)	788.98 (469.28–1129.43)	11.38 (8.27–14.56)	452.21 (328.04–580.02)
	Western Europe	0.63 (0.35–0.97)	11.08 (6.15–16.81)	0.44 (0.23–0.7)	4.85 (2.81–7.29)	15.83 (9.38–22.66)	297.19 (178.99–420.95)	10.72 (6.63–15.13)	146.18 (95.83–200.47)
	Western Sub-Saharan Africa	0.12 (0.05–0.21)	12.74 (5.61–22.25)	0.36 (0.22–0.52)	17.44 (10.42–25.78)	4.15 (1.96–6.9)	396.61 (184.07–667.7)	12.93 (8.39–18.25)	540.17 (341.5–767.06)

DALYs, disability-adjusted life-years; UI, uncertainty interval; SDI, socio-demographic index.

## Results

### The global burden of stroke attributable to HBMI

**Figure 1:**
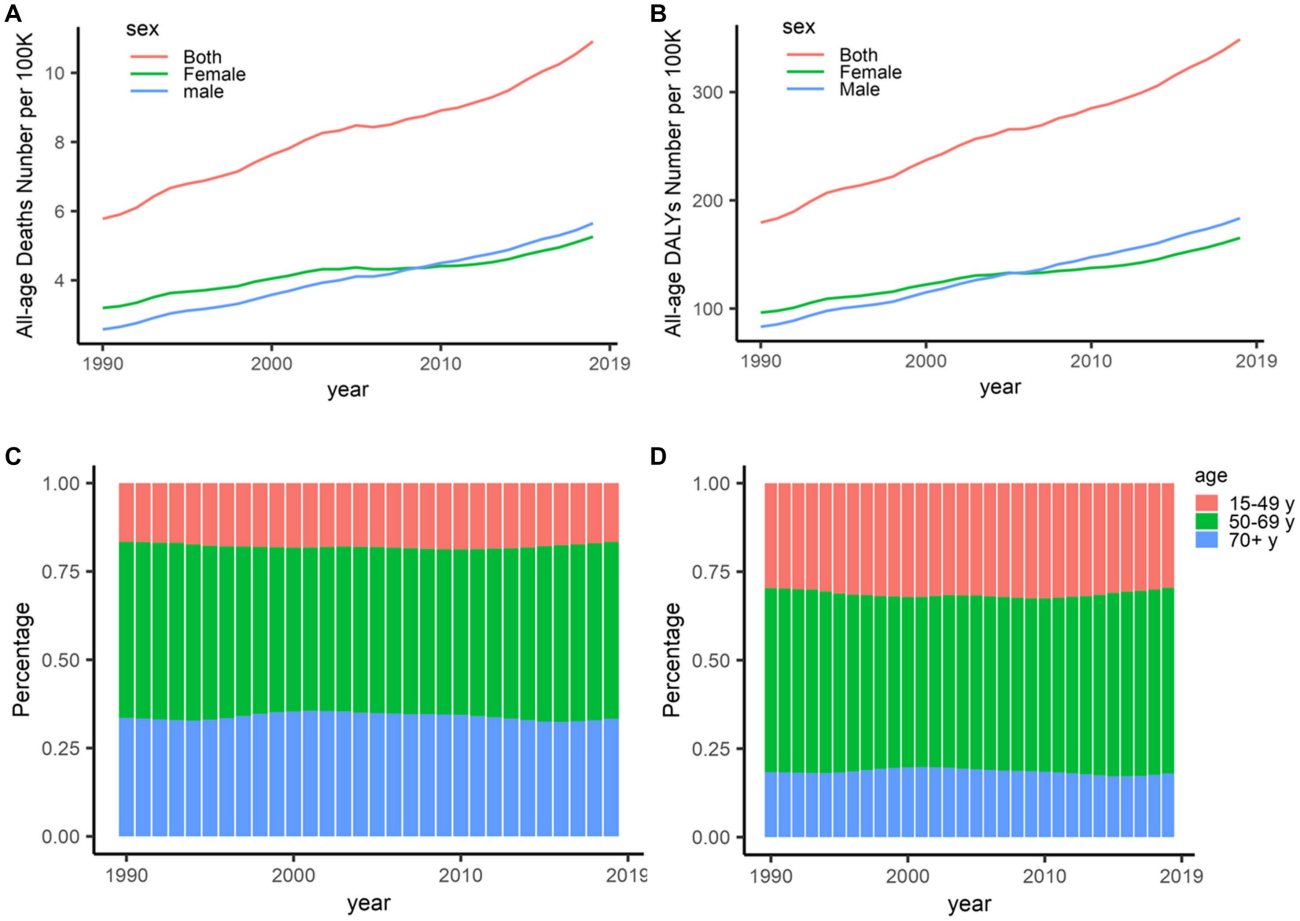
Deaths and DALYs of stroke attributable to high body-mass index, 1990–2019. **(A)** Deaths in all ages. **(B)** DALYs in all ages. **(C)** Deaths in different age distributions. **(D)** DALYs in different age distributions. DALYs, disability-adjusted life-years.

*Globally*, *deaths by stroke attributable to HBMI were on the rise from 1990 to 2019* (Figure 1A; Table 1), with an increase of 88.75%*; similarly*, *the DALYs showed rapid growth in the same times*, *with an increase of 94*.*33%*. *By gender*, *differences were shown in deaths* (*a growth of 118*.*99% for men vs a growth of 64*.*37% for women*) *and DALYs* (*a growth of 120*.*54% for men vs a growth of 71*.*69% for women*) *in 1990–2019* (Figure 1B).

Globally, the percentages of deaths and DALYs presented important differences in age. The percentages of deaths decreased among the oldest and the youngest populations but increased in middle-aged populations since 2003 (Figure 1C), which is similar to DALYs worldwide (Figure 1D). On the other hand, deaths and DALYs worldwide were on the rise among those populations aged 15–49, with an increase of approximately 25% especially for DALYs; more than half of deaths and DALYs worldwide were shared by those populations aged 50–69 in 1990–2019.

**Figure 2:**
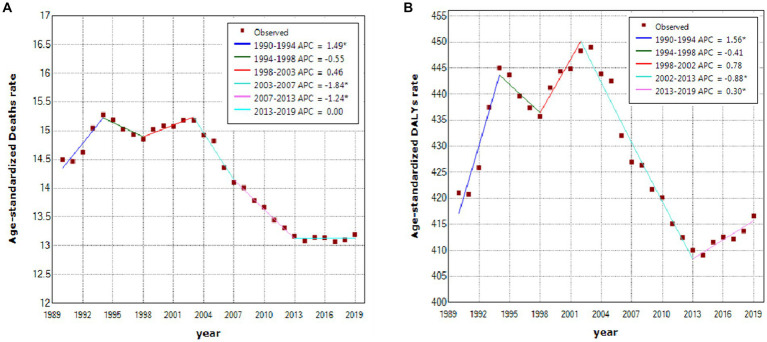
Temporal trends of stroke attributable to high body-mass index, 1990–2019. **(A)** Age-standardized deaths. **(B)** Age-standardized DALYs. ^*^Represents that the APC is significantly different at α = 0.05. DALY, disability-adjusted life-year; APC, annual percentage change.

In the past 3 decades, noticeable changes of three turning points in 1994, 1998, and 2003 took place in age-standardized deaths worldwide, with an especially decreasing trend from 2003 [15.18 per 100,000 persons (95% UI: 8.34–23.39)] to 2013 [13.17 per 100,000 persons (95% UI: 7.67–19.58)]. The APCs worldwide were − 1.84% (*p* > 0.05) in 2003–2007 and − 1.24% (*p* > 0.05) in 2007–2013 (Figure 2A). Globally, age-standardized DALYs overall were on the rise in 1990–2002 but declined in 2003–2013; the highest APC in 1990–1994 (APC = 1.56%, *p* < 0.05), a significant APC in 2002–2013 (APC = −0.88%, *p* < 0.05), and a turning point in 2013 with an increasing trend since then (APC = 0.30%, *p* < 0.05) was observed (Figure 2B).

### The national burden of stroke attributable to HBMI

**Figure 3:**
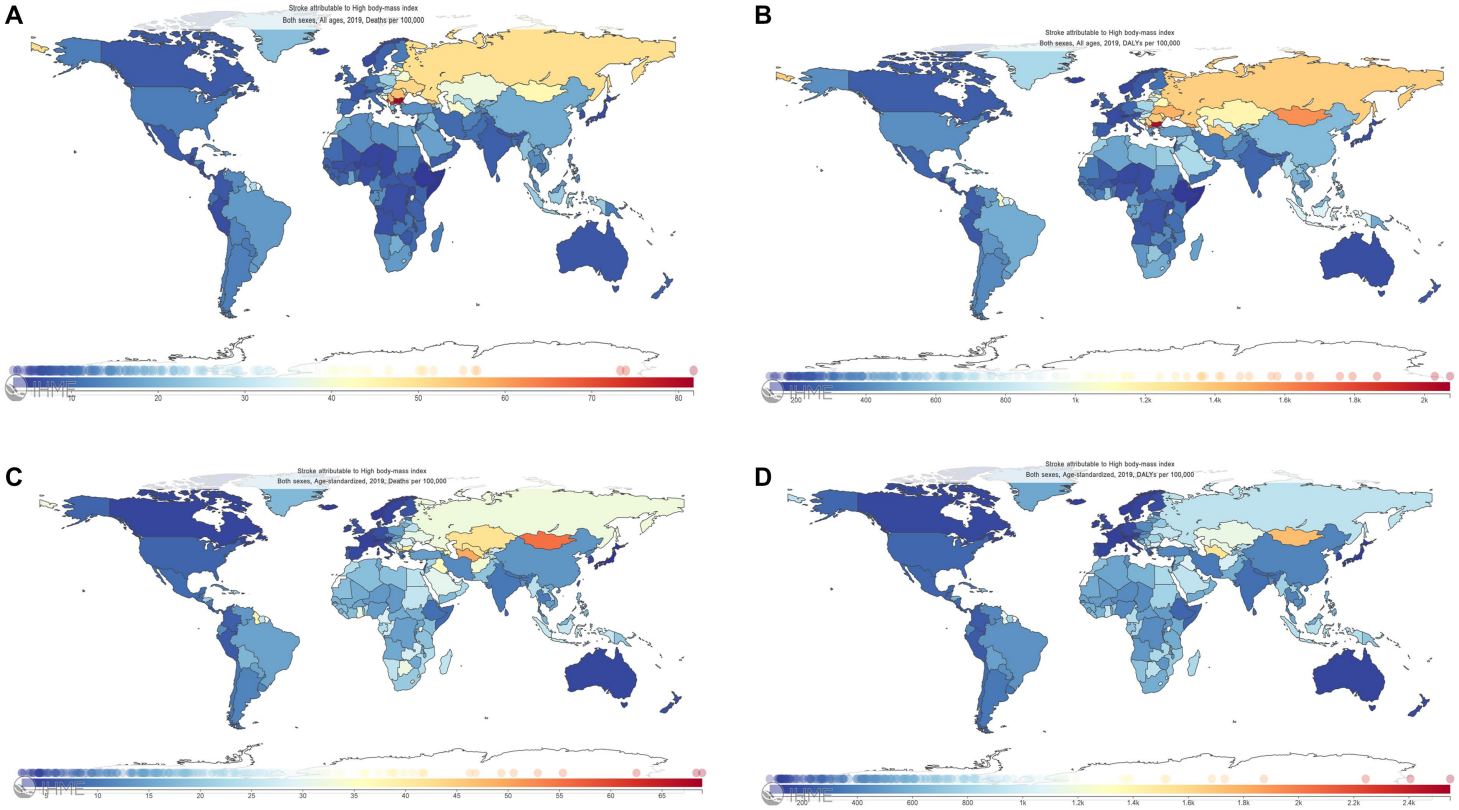
Deaths, DALYs, age-standardized deaths, and age-standardized DALYs of stroke attributable to high body-mass index among 204 countries and territories, 2019. **(A)** Deaths. **(B)** DALYs. **(C)** Age-standardized deaths. **(D)**: Age-standardized DALYs. The Global Burden of Disease Project 2019, Institute for Health Metrics and Evaluation, University of Washington, Seattle, WA, United States. DALYs, disability-adjusted life-years.

Among 204 countries and territories in 2019 (Figures 3A–D), China had the highest number of deaths (0.27million, 95% UI: 0.12–0.45 million); India, the Russian Federation, Indonesia, and the United States followed; on the contrary, Tokelau, Niue, San Marino, Nauru, and Tuvalu had the five lowest deaths in sequence. Similarly, China had the leading DALYs (8.19 million, 95% UI: 3.89–13.43); India, Indonesia, the Russian Federation, and the United States followed in 2019. Ten countries and territories had an age-standardized death rate > 50 per 100,000 persons in 2019, wherein the highest age-standardized death was observed in Bulgaria [81.73 per 100,000 persons (95% UI: 48.49–122.60)]. In all countries and territories, age-standardized DALYs in 2019 were more than 100 per 100,000 persons, wherein Kiribati had the highest level [2,073.31 per 100,000 persons (95% UI: 1,333.41–2,925.71)].

### The regional and SDI burdens of stroke attributable to HBMI

**Figure 4:**
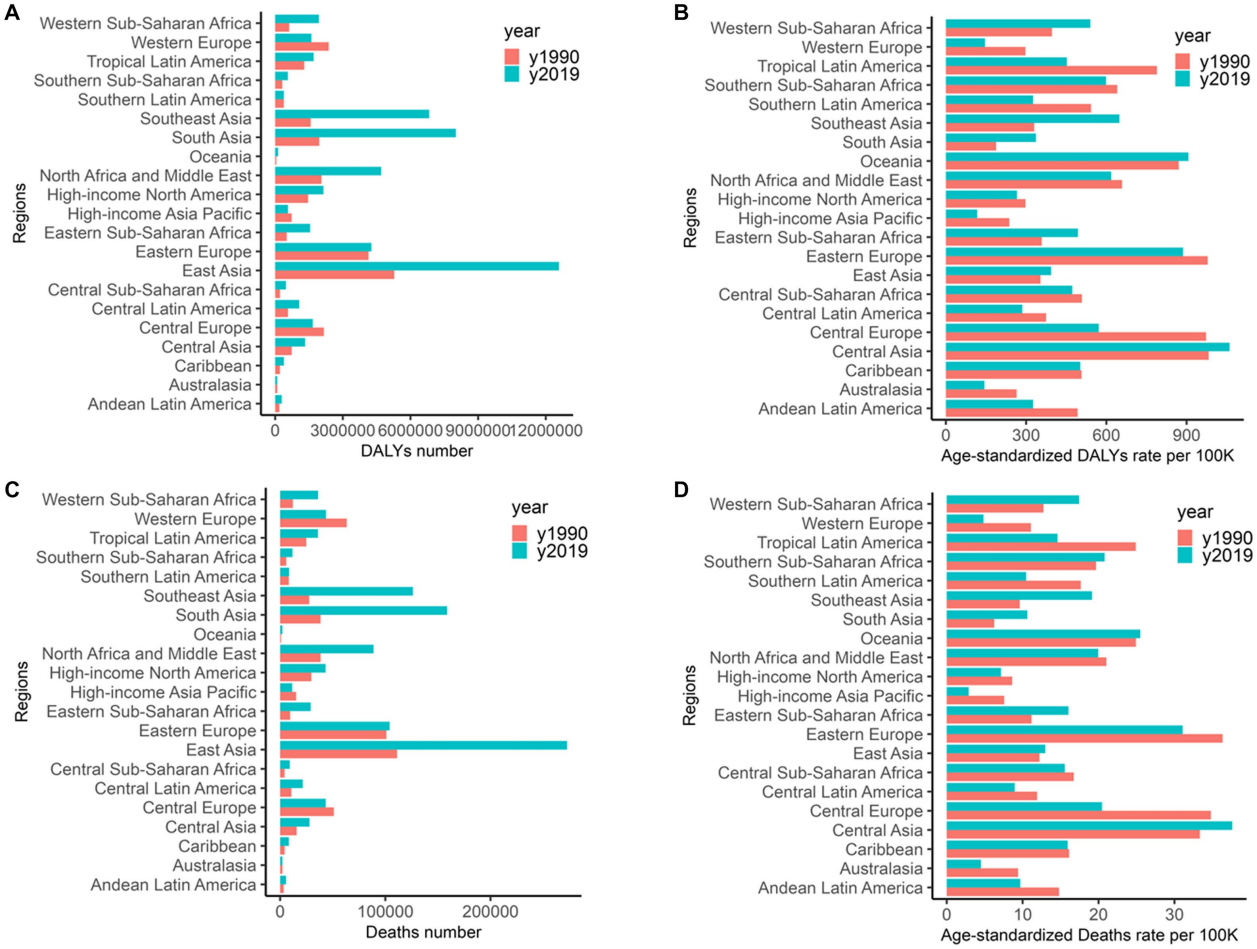
Deaths, age-standardized deaths, DALYs, and age-standardized DALYs of stroke attributable to high body-mass index in 21 regions. **(A)** DALYs. **(B)** Age-standardized DALYs. **(C)** Deaths. **(D)** Age-standardized deaths. DALYs: disability-adjusted life-years.

*Important differences in trends were found among different GBD regions*. East Asia had the highest deaths in 1990 and 2019, with 0.11 million (95% UI: 0.03–0.24) in 1990 and 0.27 million (95% UI: 0.12–0.47) in 2019, and with an increase of 245.45% for deaths and 238.18% for the DALYs, respectively; *Central Asia had the highest age-standardized deaths* [37.61 per 100,000 persons (95% UI: 24.48–51.76)] in 2019; Eastern Europe had the highest age-standardized deaths [36.34 per 100,000 persons (95% UI: 22.95–50.71)] in 1990. Among 21 regions, Central Asia had the highest age-standardized DALYs in 1990 [982.54 per 100,000 persons (95% UI: 641.88–1,327.28)] and in 2019 [1,060.70 per 100,000 persons (95% UI: 735.10–1399.77); Table 1; Figure 4].

**Figure 5:**
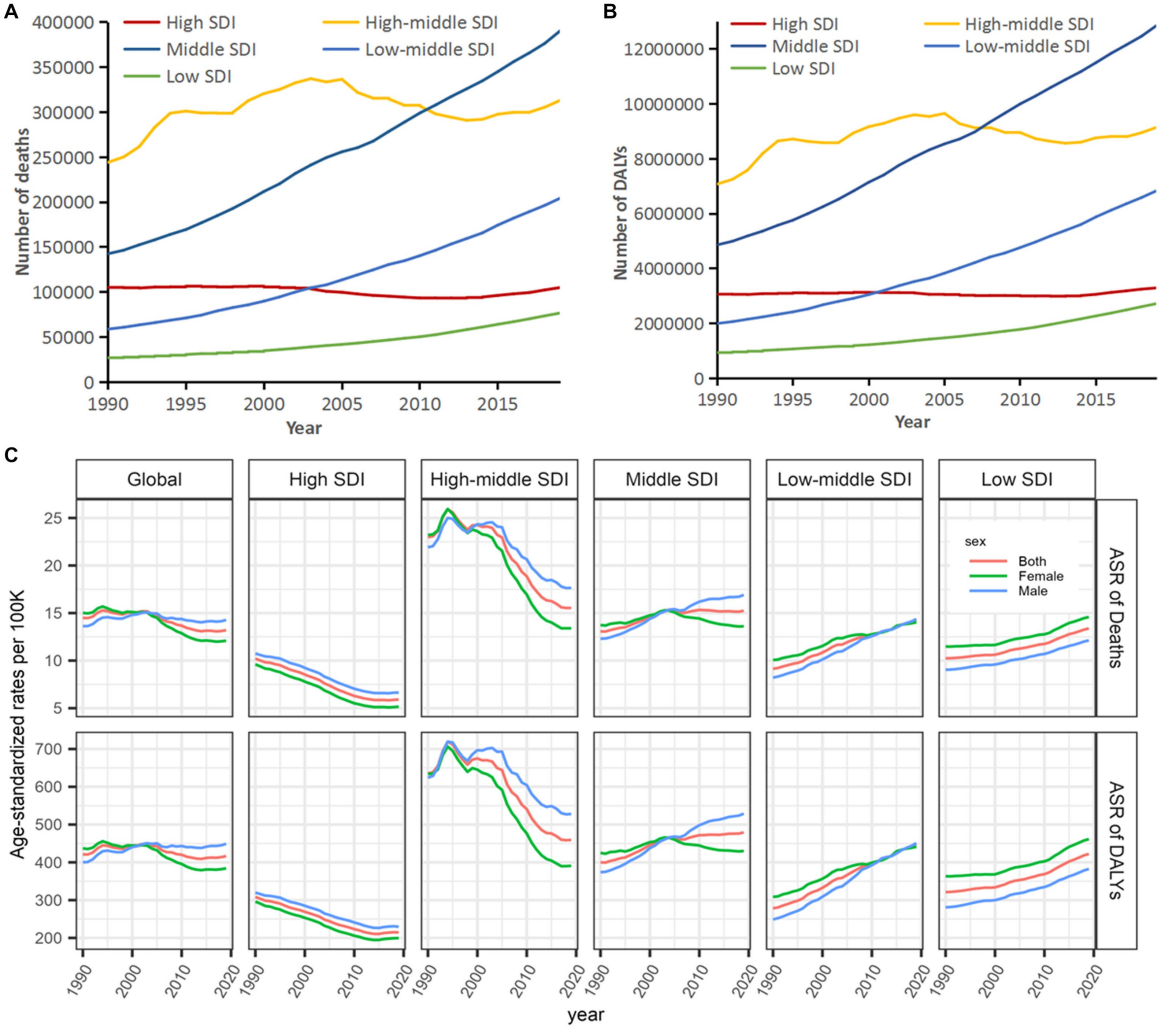
Tendencies in deaths and DALYs of stroke attributable to high body-mass index, 1990–2019. **(A)** Deaths. **(B)** DALYs. **(C)** Age-standardized deaths and DALYs. SDI, socio-demographic index; ASR, age-standardized rate; and DALYs, disability-adjusted life-years.

Additionally, significant geographical variations in the burden of stroke attributable to HBMI by the GBD regions were noted. Numbers of deaths and DALYs in different SDI regions showed great disparities in the past 3 decades, with the lowest numbers of deaths and DALYs in low SDI regions, a flattening off in high and high-middle SDI regions, and a low and steady level, in particular, in high SDI regions. *High-middle SDI regions shared the highest numbers of deaths and the DALYs before 2011*, *but these numbers were surpassed by middle SDI regions since then and rapid growth was experienced*. Almost two-thirds of the global burdens were borne by high-middle to middle SDI regions (Figures 5A,B). For global tendencies of age-standardized rates, DALYs and deaths, respectively, showed a flattening off and a slight decline in 1990–2019, *wherein the lowest age-standardized rates of DALYs and deaths were prevalent in high SDI regions*, *and these two rates decreased from high-middle to low SDI in the other four regions*, *interestingly*, *with a decreasing trend in high and high-middle SDI regions but an increasing trend in the middle*, *low-middle*, *and low SDI regions*. Meanwhile, age-standardized rates of deaths and DALYs in high and high-middle SDI regions were higher in men than those in women; conversely, these two rates in middle, low-middle, and low SDI regions were lower in men than those in women (Figure 5C).

### Associations of the SDI with age-standardized deaths and DALYs

**Figure 6:**
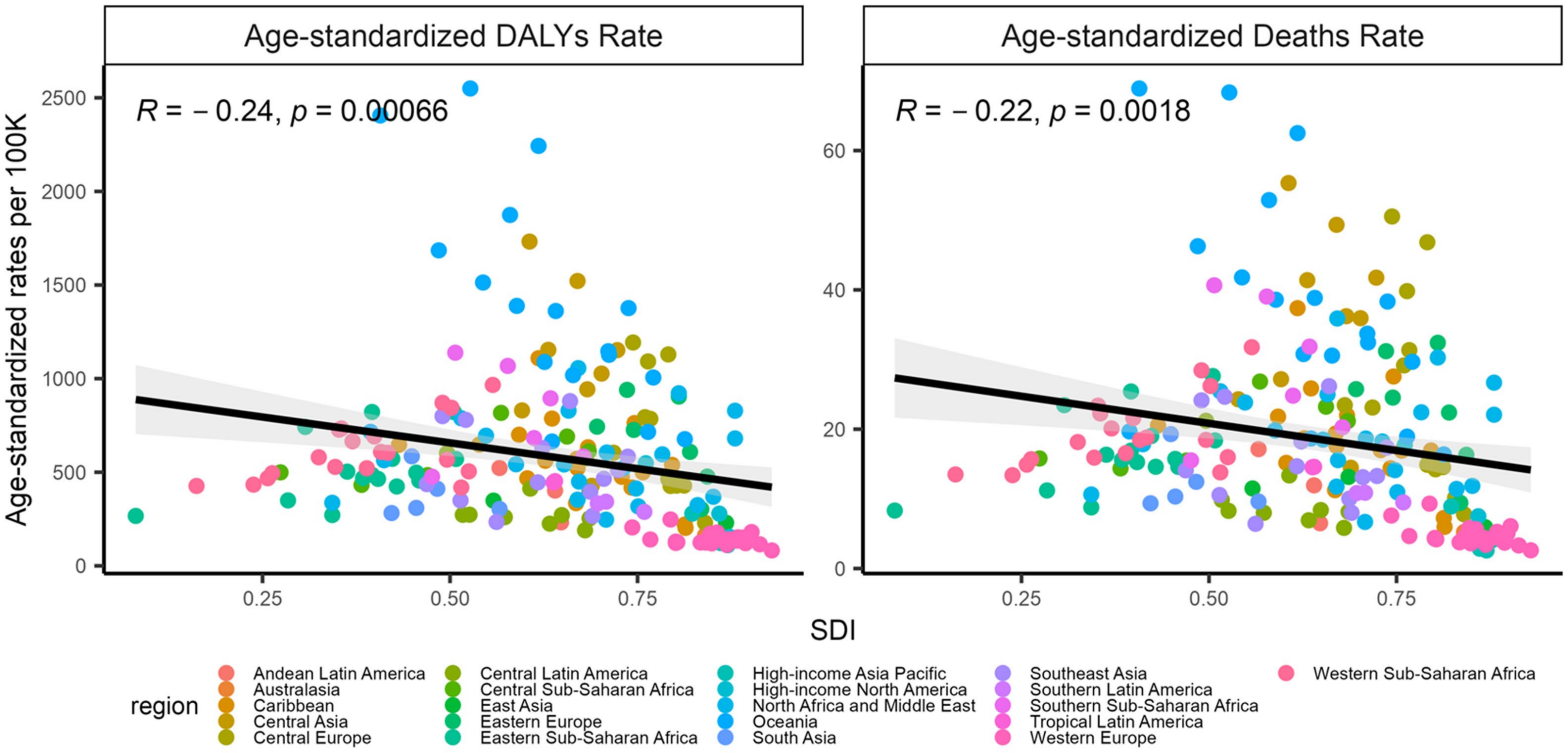
Associations of the SDI with age-standardized DALYs and deaths, 2019. The left: SDI and age-standardized DALYs; the right: SDI and age-standardized deaths. The points represent countries or territories. The solid line color blue represents expected values across the SDIs; the shadow represents the 95% confidence intervals; the Pearson correlation coefficients and the *p* values were denoted. SDI, socio-demographic index.

Figure 6 shows *that the SDI was associated with age-standardized rates of deaths and DALYs*, *with a significance for age-standardized DALYs* (*R = −0*.*24*, *p < 0*.*001*) *and age-standardized deaths* (*R = −0*.*22*, *p = 0*.*0018*) *in 2019*.

## Discussion

This study showed more than a 1.89-fold increase in deaths and a 1.94-fold increase in DALYs from global stroke attributable to HBMI in 1990–2019 although their age-standardized rates had a slight decline, with 8.90% for age-standardized deaths, and 1.06% for age-standardized DALYs. Our results suggest that the immense financial burden caused by stroke attributable to HBMI worldwide should have deteriorated in recent decades (17–19).

In this work, different analytical models were performed among different age groups for the percentages of deaths and DALYs. For death by age, its percentages showed to be 33.27% for those populations aged >70, 50.08% for those populations aged 50–69, and 16.65% for those populations aged 15–49; similarly, *DALYs showed the percentages to be 17*.*94% for those populations aged approximately 70*, *52*.*49% for those populations aged 50–69*, *and 29*.*57% for those populations aged 15–49*; such results revealed that those populations aged 50–69, which is a high incidence age for stroke, shared main contributions to deaths and DALYs of stroke attributable to HBMI. *Our results are consistent with the fact that the incidence of stroke worldwide was increasing in contrast to older adults* (20–23) *and HBMI was associated with a higher risk of stroke among young adults* (24). *Those populations who were aged 15–49 and suffered from stroke were apt to have high DALYs but not deaths besides the other outcomes such as increasing disabilities although stroke is the most common cause of complex disabilities* (25) *and functional disability among stroke survivors*, *frequently affecting basic self-care activities* (26, 27). *We found that DALYs was in line with the trend for HBMI among those young populations*, *which shows that stroke leads to a long-term disability with far-reaching effects on the quality of life of patients and their caregivers* (21, 28).

In actual distributions of the APC and age-standardized rates or the fitted trends (both deaths and DALYs) for stroke attributable to HBMI, a significant increasing trend in the APC but a decreasing trend in age-standardized rates (both deaths and DALYs) occurred in 1990–2003 although this profile has improved since 2003; globally, *age-standardized deaths with a flatter trend in 2003*–*2013 and age-standardized DALYs with a moderate upward trend since 2013 were mainly contributed to by* Central Asia, Oceania, Eastern Europe, etc., *which may be due to HBMI growth*, *cognitive developments*, *and the improvements to emergency treatments and recovery*, *and the treatments for complications of a stroke*. *Additionally*, *deaths and DALYs decreased after age intensity was removed although an increasing trend in deaths by stroke attributable to HBMI was observed*.

Trends in deaths and DALYs by stroke attributable to HBMI remained upward across the SDI regions, with a decline in high and high-middle SDI regions, especially with a low level in high SDI regions but an increasing trend in middle and low-middle SDI regions, which reveals that high SDI regions are paying more attention to health problems by HBMI for preventing and controlling stroke. Deaths and DALYs by stroke attributable to HBMI deteriorated in middle and low-middle SDI regions, with fewer means and insufficient payments to prevent and treat this disease on a large scale in fast-increasing and increasingly aging populations (29); middle and low-middle SDI regions had a greater prevalence or more effect of HBMI on stroke than high and high-middle SDI regions (30); *such previous results highlight an inadequacy in primary preventive efforts worldwide* (31, 32)*; contrarily*, *high SDI areas have developed medical standards*, *timely treatments*, *and sufficient financial resources to launch plans and initiatives aimed at promoting healthy food* (*usually more expensive than “junk food”*) *for lowering obesity occurrence*, *while medium and advanced middle to high SDI regions have a large population but poor acute healthcare and awareness of stroke*, *which naturally causes the most deaths and highest DALYs caused by stroke attributable to HBMI*. *Therefore*, *inequality services for the prevention and treatment in lower SDI regions have become an immense barrier to lower stroke attributable to HBMI*.

Body mass index, which is one of the best-known measurements for body size (33), was assessed in this work. Although it is a simple and crude method with several limitations, such as its inability to capture a distribution between lean body mass and adipose tissues, the BMI is a better predictor of cardiovascular diseases and shows a curvilinear (U-shaped) association with mortality risk (34). The other anthropometric measurements and their associations with obesity have been studied, wherein each shared its own strengths and limitations (35, 36). *We also observed dissimilar burdens of the disease in developing and developed countries; hence*, *we are considering doing some analyses in the future*. *This study was comprehensive in its global coverage and is the largest stroke epidemiological dataset to date*, *and it tried*, *first*, *to provide systematic estimates of the burden of stroke attributable to HBMI*. *However*, *some limitations are included*. *First*, *a scarcity of high-quality epidemiological data from different countries limited studies of both high and low methodological qualities from the GBD countries*. *Second*, *there was substantial regional heterogeneity in the study data*, *and different countries might account for some differences noted in the epidemiological characteristics of the disease and disease burden*. *Nevertheless*, *we applied standard methodological criteria for the studies across the past 3 decades to avoid marked effects on the estimates by the above limitations; hence*, *we believe that the quality of data was consistent for the included studies*.

## Conclusion

*In this study*, *we showed that global burdens of stroke attributable to HBMI were continuously on the rise*. *Therefore*, *it is necessary to insist on avoiding overweight and obesity throughout the lifespan*, *which also foreshadows a great challenge worldwide to establish causes of disparities and changes in trends in the burden of stroke attributable to HBMI among countries of different income levels*. *Findings from this study will also help with the development and monitoring of the effectiveness of stroke prevention and management and rehabilitation strategies in different countries and among different populations*.

## Data availability statement

Publicly available datasets were analyzed in this study. This data can be found here: Institute for Health Metrics and Evaluation (IHME), Global Health Data Exchange (GHDx), and Global Burden of Disease (GBD) study, http://ghdx.healthdata.org/gbd-results-tool.

## Author contributions

XG, JL, XY, and QZ made contributions to the acquisition, interpretation, and analysis of data. FZ and ZZ designed the study. FZ drafted and revised the article. All authors contributed to the article and approved the submitted version.

## Funding

This work was supported by the Guangzhou Municipal Science and Technology Project (201704030132, 202102080467, and SL2022A03J00151) and the Guangdong Medical Research Foundation (A2022209). The funders had no role in the study design, data collection or analysis, or preparation of the manuscript.

## Conflict of interest

The authors declare that the research was conducted in the absence of any commercial or financial relationships that could be construed as a potential conflict of interest.

## Publisher’s note

All claims expressed in this article are solely those of the authors and do not necessarily represent those of their affiliated organizations, or those of the publisher, the editors and the reviewers. Any product that may be evaluated in this article, or claim that may be made by its manufacturer, is not guaranteed or endorsed by the publisher.

## Footnotes

^1^ healthdata.org

## Abbreviations

BPD: Bipolar disorder
GBD: Global burden of disease
DALYs: Disability-adjusted life-years
ASR: Age-standardized rate
